# 

**Published:** 1898-03

**Authors:** 


Vol. LII. MARCH, 1898. No. 3.

### THE DENTAL REGISTER

LIBRARY
SURGEON GENERAL'S OFFICE
MAR.-[illegible]-1898

A MONTHLY JOURNAL.

Devoted to the Interests of the Profession.

EDITED BY

J. TAFT, D. D. S.

PUBLISHED BY
SAM'L A. CROCKER & CO.,
OHIO DENTAL AND SURGICAL DEPOT,
35, 37 and 39 West Fifth Street, CINCINNATI, OHIO.

Entered at the Postoffice at Cincinnati, O., as second class mail matter.

TERMS: $2.00 PER ANNUM IN ADVANCE. SINGLE COPIES, 25 CTS.

